# Comparison of extended anterolateral approach in treatment of simple/complex tibial plateau fracture with posterolateral tibial plateau fracture

**DOI:** 10.1186/s13018-018-1007-7

**Published:** 2018-11-28

**Authors:** Liangjun Jiang, Qiang Zheng, Zhijun Pan

**Affiliations:** The Orthopedics Department of 2nd Affiliated Hospital of Medical, College of Zhejiang University, The Jiefang road 88#, Hangzhou, Zhejiang China

**Keywords:** Posterolateral tibial plateau fracture, Extended anterolateral approach, Arthroscopy treatment, Fracture reduction and fixation

## Abstract

**Background:**

Our hospital has recently used the extended anterolateral approach in posterolateral tibial plateau fracture. We compared the clinical effects of this method in Schatzker type II or type V/VI fractures with posterolateral tibial plateau fracture based on our patients.

**Methods:**

The patients from January 2013 to December 2015 were summarized, and some of them were assisted with arthroscopy. According to Schatzker classification, patients with Schatzker type II fracture were divided into group A; patients with Schatzker type V/VI fracture were divided into group B. The fracture characteristics, operation statistics, and postoperative functional evaluation of each group were compared.

**Results:**

A total of 46 patients were included in the study and were followed up for 23–45 months. There were 24 cases in group A and 22 cases in group B. The operation time and the amount of bleeding were significantly less in group A (*P* < 0.05). Twelve cases were assisted with arthroscopy including 6 patients in each group. The fracture healing time made no significant difference in the two groups (*P* > 0.05). All patients experienced no significant influence on daily life. The knee Rasmussen score was 26.8 in group A and 23.5 in group B (*P* > 0.05), and the knee range motion was 115.5° in group A and 106.6° in group B (*P* > 0.05). The excellent and good rate of reduction was 91.7% in group A and 81.8% in group B (*P* > 0.05), but the excellent rate of reduction was 83.3% in group A and 27.3% in group B (*P* < 0.05). The unfixed rate of posterolateral fracture was 16.7% in group A and 36.4% in group B (*P* > 0.05). One patient in group B suffered postoperative wound infection.

**Conclusions:**

The extended anterolateral approach could obtain similar satisfactory clinical results in simple/complex tibial plateau fracture with posterolateral tibial plateau fracture. It seemed that easier operation, better posterolateral fracture reduction, and fixation occurred in relative simple fracture from our cases.

**Trial registration:**

It was a retrospective study. This study was consistent with the ethical standards of the Second Affiliated Hospital of Zhejiang University Medical College and was approved by the hospital ethics committee and the trial registration number of our hospital was 20170053.

## Background

A posterolateral fracture fragment in tibial plateau was defined as any separate posterolateral quadrant-based articular fracture fragment, with the extension of the fracture line to the posterolateral cortex [1]. This type of fracture was not as uncommon as previously believed. Either isolated or combined with another tibial plateau quadrant, its incidence ranged from 7 to 15% in tibial plateau fractures [2, 3]. The unreduced posterolateral tibial plateau fracture would lead to knee flexion instability and activity abnormalities [4, 5]. However, the reduction and fixation of posterolateral tibial plateau fracture were difficult, and there was no standard approach in clinical practice. Luo et al. used an inverted L-shaped incision [2, 6]; Chang et al. used a single posterolateral approach to expose and reduce the fracture directly [7, 8]; Lobenhoffer et al. firstly used the fibular osteotomy approach in the treatment of posterolateral fracture [9, 10]. However, these shortcomings such as complex operation technique, possibility of damage to important blood vessels and nerves, and trouble with changing the body position existed in these approaches. Currently, the extended anterolateral approach was developed for posterolateral tibial plateau fracture [11, 12].

The extended anterolateral approach had the advantages of simple operation technique, little surgical damage, easy body position during operation, and easy removal of the internal fixation latterly. However, it also had some shortcomings. One shortcoming was that there were some difficulties existed in the exposure and reduction of comminuted posterolateral fracture. If the tibial plateau fracture was simple, it was easy to be exposed and reduced. However, it was difficult to judge whether the posterolateral fracture was anatomically reduced by C-arm X-ray or direct vision in the comminuted fracture. Arthroscopy treatment could directly expose the articular surface and especially had advantages in comminuted tibial plateau fracture. It had become an important assisted technology due to its advantages of little operation damage, confirmed reduction of the articular surface, and ability to repair meniscus and ligaments [13, 14]. So, it might be helpful in the treatment of posterolateral tibial plateau fracture. Another shortcomings of the extended anterolateral approach was impossible to place buttress plates posteriorly, and the anterolateral tibial locking plate with rafting screws might not provide enough mechanical stability to comminuted posterolateral fracture. Some comminuted fracture fragments might not be fixed by rafting screws. Therefore, the reduction and fixation stability might be different in simple/complex tibial plateau fracture with posterolateral tibial plateau fracture in the extended anterolateral approach treatment.

Though the extended anterolateral approach or arthroscopy treatment was widely used in the tibial plateau fracture, there was no research to compare the extended anterolateral approach assisted with arthroscopy treatment in simple/complex tibial plateau fracture with posterolateral plateau fracture. Currently, the differences of operation techniques and clinical results between simple/complex tibial plateau fractures were unknown. We retrospectively analysed patients with posterolateral tibial plateau fracture who were divided into two groups by different fracture types in recent years in our hospital. We summarized the experience and compared the clinical results in these two groups to confirm the differences in simple/complex tibial plateau fracture with posterolateral tibial plateau fracture by this method. We hope our findings can be used to improve the treatment of posterolateral tibial plateau fracture.

## Materials and methods

### Patients

We retrospectively analysed the patients with posterolateral tibial plateau fracture treated by the extended anterolateral approach at our hospital between January 2013 and December 2015. The exclusion criteria were as follows: open fracture, > 3 weeks between the injury and the initial operation, presence of a pathological fracture, vascular or nerve injury, and knee arthritis history.

A total of 46 patients were included in the study and were followed up for 23–45 months (mean, 31.9 months). Twenty patients were male, and 26 were female, aged 29–77 years, with an average of 53.9 years. According to Schatzker classification, 24 patients of Schatzker type II fracture were divided into group A, and 18 patients of type V fracture and 4 patients of type VI fracture were divided into group B. According to the three-column classification, 6 cases involved the posterior column, 18 cases involved the lateral and posterior columns, 6 cases involved the medial and posterior columns, and 16 cases involved three columns. The causes of injury were tumbles in 28 cases (60.7%), traffic accidents in 14 cases (30.4%), falls in 2 cases (4.3%), and direct impact in 2 cases (4.3%). The injury mechanism was flexion valgus injury in 30 cases (65.2%), flexion varus injury in 4 cases (8.7%), extension injury in 6 cases (13%), and flexion injury in 6 cases (13%). Twenty-two patients had a fibular fracture, and 6 had associated injuries (3 vertebral fractures, 1 skull fracture and pelvic fracture, 1 pelvic fracture, and 1 ankle fracture) (Table 1).

**Table 1:** Patient demographics

Demographic	
Cases	46
Average age (years)	53.9
Follow-up time (months)	31.9
Gender	
Male	20
Female	26
Cause of injury	
Tumbles	28 (60.7%)
Traffic	14 (30.4%)
Falls	2 (4.3%)
Direct impact	2 (4.3%)
Schatzker classification	
II	24
V	18
VI	4
Three-column classification	
Posterior column	6
Lateral and posterior columns	18
Medial and posterior columns	6
Three columns	16
Injury mechanism	
Flexion valgus	30 (65.2%)
Flexion varus	4 (8.7%)
Extension	6 (13%)
Flexion	6 (13%)
Associated injury	
Fibular fracture	22
Vertebral fracture	3
Skull fracture	1
Pelvic fracture	2
Ankle fracture	1

### Preoperative planning

Routine preoperative examinations consisted of plain radiography and CT scan. The injured limb was fixed with plaster or calcaneal pin traction temporarily, and the surgical treatment time was based on the condition of the soft tissue. The injury mechanism, surgical approach, and treatment method of the fracture were determined by the fracture characteristics [15].

### Surgical treatment

We set the patient in the supine position on an ordinary operation table. In group A, we usually used a single extended anterolateral approach to reduce and fix the lateral plateau fracture with a posterolateral fracture. In group B, the tibial plateau fracture included the medial and lateral plateaus’ fractures, so we used two incisions consisted of a medial incision and an extended anterolateral approach to make the treatment. In group B, we did the medial incision first. Usually, the medial plateau fracture was a metaphyseal fracture, and the articular surface was intact without any compression; there was no need to open the medial joint capsule. We only need to make sure that the medial tibial cortex is anatomically reduced. After the X-ray confirmed that the medial plateau was reduced, we used a pre-bending reconstruction plate (Synthes GMBH, Zuchwil, Switzerland) to fix the medial tibial plateau, and then the following extended anterolateral approach would be made to reduce and fix the lateral plateau fracture with a posterolateral fracture.

We made the extended anterolateral approach as follows. Firstly, we put the knee joint in modest flexion with a bump underneath the knee joint. A 10- to 15-cm-long curvilinear incision centred over Gerdy’s tubercle was made. After the skin incision, subcutaneous dissection was made approximately 2 cm along the skin incision. Then, a fascial incision was made along the same line as the skin incision. The iliotibial band was split in the middle along the direction of the fibres and sharply elevated from Gerdy’s tubercle anteriorly and posteriorly. Then, the fascial incision was extended down to the crural fascia. The dissection was extended posteriorly by taking down the extensor muscles from the lateral surface of the lateral plateau to the point right in front of the fibular collateral ligament (FCL). By this point, we flexed the knee joint further up to 90° to relax the fibular collateral ligament and the common peroneal nerve. Then, a retractor was placed on the inferior part of the FCL to retract the FCL posterolaterally. The interval between the FCL and the posterolateral surface of the lateral plateau (para-FCL space) was developed by dissecting loose soft tissues from the posterolateral plateau. If we did not use arthroscopy treatment, we directly opened the joint capsule and retracted the lateral meniscus superiorly to make visualization of the joint surface. If we used arthroscopy treatment, we made two arthroscopy incisions and did not open the articular capsule, and then we made the visualization of the joint surface with the arthroscopic monitor.

The next step depended on the type of fracture being treated. When the fracture involved compression of the joint surface and a displaced lateral split, the split portion of the fracture was opened like a book to gain access to the compressed fragments. These fragments were then reduced to the joint surface. After the C-arm X-ray checked the reduction, we used Kirschner wires for temporary fixation. Then, the split was closed, reduced, and lateral locking plate (Synthes GMBH, Zuchwil, Switzerland) was placed. The plate was placed as posteriorly as possible and the transverse arm stretched to the supra-fibular-head space. In such situation, at most two rafting screws can support the articular surface of the posterolateral column. When a split fracture was nondisplaced or hinging it open would require extensive dissection of the intact bone and periosteum, we used the “open-window and reduction rod” technique for fracture reduction. We always used this method when arthroscopy was assisted. A cortical window was created on the anterolateral tibial metaphysis 3–5 cm below the articular surface. The depression of the articular surface could be elevated using a rod to restore the congruence of the articular surface. We confirmed the fracture was reduced on the C-arm X-ray or arthroscopic monitor. After reduction, Kirschner wires and then the plate (Synthes GMBH, Zuchwil, Switzerland) were placed as previously introduced. We would check reduction quality, implant location, and screw lengths by C-arm X-ray or arthroscopic monitor at the end of surgery again.

### Postoperative treatment

**Fig. 1:**
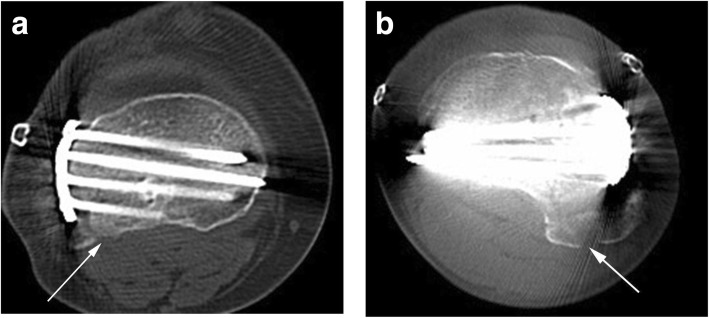
The CT scan used to evaluate the posterolateral fracture fixation. **a** Cross CT scan showed screws fixation of posterolateral tibial plateau fracture, the arrow indicated posterolateral fracture was completely fixed, no free bone fragment; **b** Cross CT scan showed only part of the posterolateral fracture was fixed, the arrow indicated one bone fragment was free

Postoperative drainage routinely continued for 24 h, and antibiotics were routinely administered for 48 h to prevent infection. Passive joint function activity was performed immediately after the operation, and partial weight training was started 2 weeks after the operation under the doctor’s guidance. Patients were followed up every 3 months, and knee joint pain, joint activity, and plain radiography were tested each time. The operation time, blood loss, fracture healing time, fracture reduction, knee function score, knee range motion, and postoperative complications of each patient were noted. According to the standard clinical and radiological criteria to judge the healing time of the fracture, the Rasmussen score system [16] was used to evaluate knee joint function, and the method proposed by Biggi et al. [17] was used to evaluate fracture reduction. Whether the posterolateral fracture fragment was fixed was evaluated by CT scan (Fig. 1).

### Statistical analysis

The operation time, blood loss, fracture healing time, knee Rasmussen score, and range motion were compared between group A and group B by the independent *t* test. The fracture reduction and the posterolateral fracture fragment fixation were compared between group A and group B by the chi-square test (Fisher’s exact test). *P* < 0.05 was considered significant. SPSS software (22nd edition, SPSS, Chicago) was used to record and analyse the study results.

## Results

### Operation statistics and functional evaluation

**Fig. 2:**
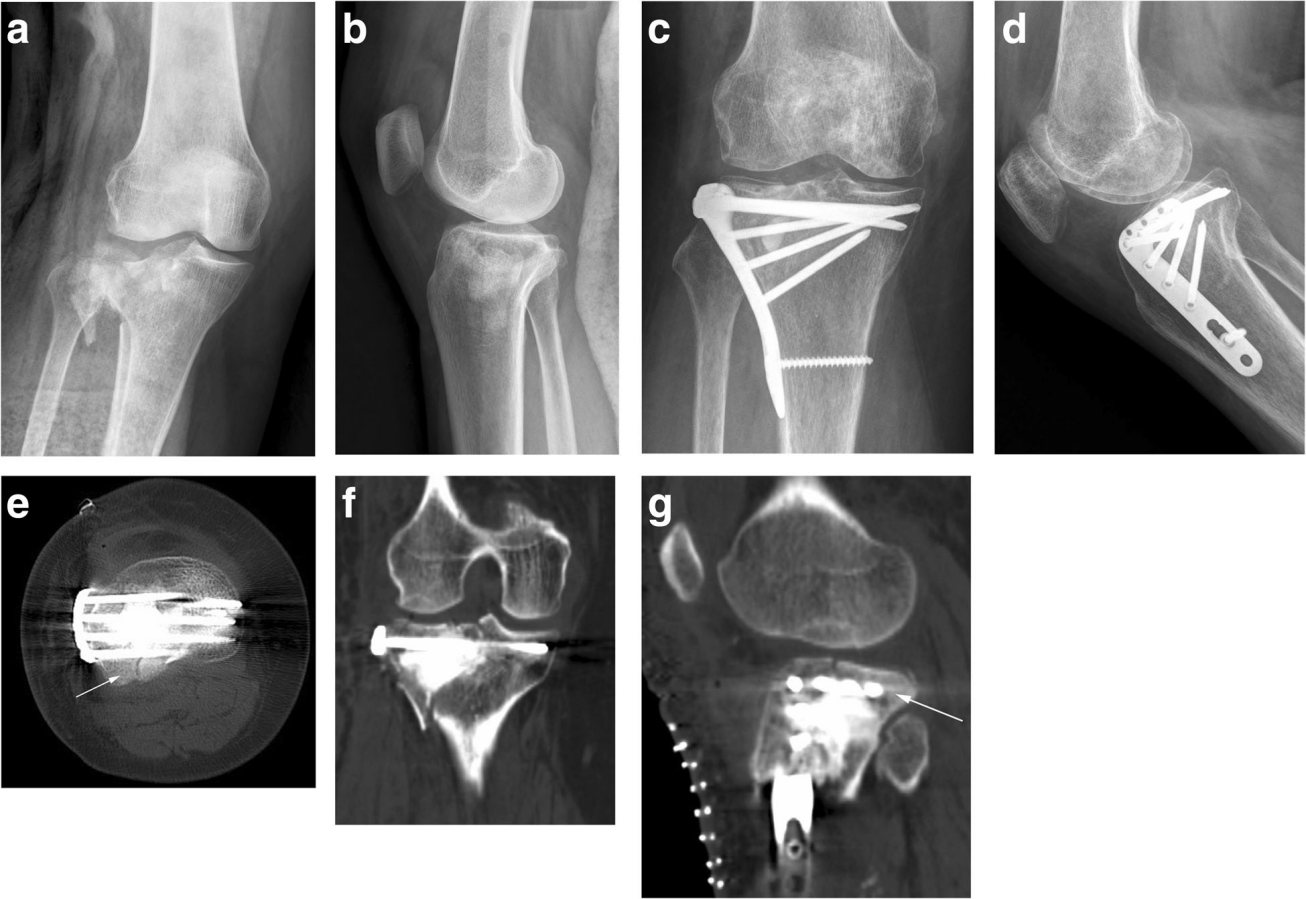
A case of type II tibial plateau fracture, female, 60 years old. **a**, **b** Preoperative X-ray showed lateral plateau fracture, increased width of the plateau, and posterolateral fracture. **c**, **d** Postoperative X-ray showed that the fracture was anatomically reduced and fixed by a lateral locking plate with rafting screws. **e**–**g** CT scan showed an anatomical reduction, and the arrow indicated the posterolateral fracture got satisfactory fixation by screws

**Fig. 3:**
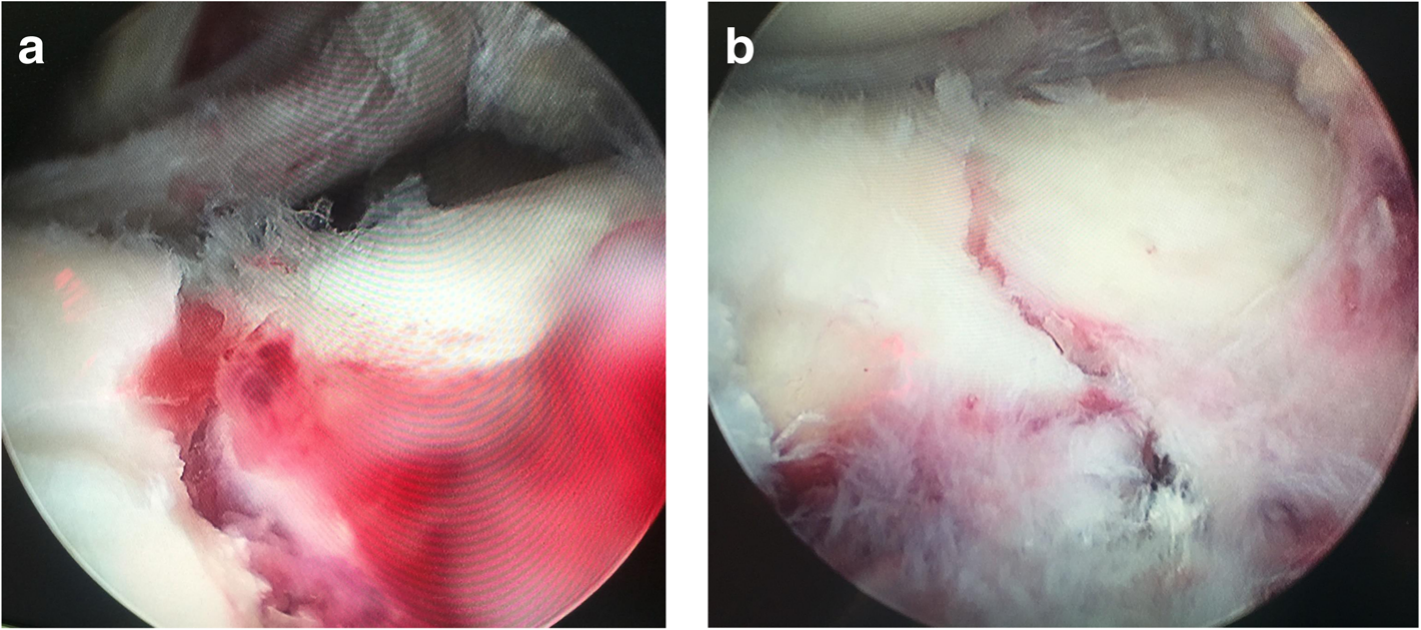
Findings during arthroscopy treatment. **a** The articular cartilage was broken and displaced before reduction. **b** The articular cartilage was reduced by open-window rod technique, and it was smooth

The operation time was 55–150 min (average 124 min) in group A and 110–300 min (average 175 min) in group B; the amount of bleeding was 20–400 ml (mean 118 ml) in group A and 50–500 ml (mean 190 ml) in group B. Both made significant differences (*P* < 0.05). In group A, patients were treated with a single anterolateral plate (Fig. 2); In group B, patients were treated with medial and lateral plates. Twelve patients received arthroscopy treatment (Fig. 3) including six patients in each group. Patients with lumbar fractures were treated conservatively, patients with ankle fracture were treated with surgery, and patients with pelvic fractures underwent staged surgery. All patients had achieved bony union at the last follow-up. The average fracture healing time was 4.58 months (3–6 months) in group A and 5.54 months (4–8 months) in group B, and there was no significant difference (*P* > 0.05). Fourteen patients removed the internal fixation at the last follow-up.

All patients experienced no significant influence on daily life. They could walk on the floor and up and down the stairs and engage in light physical labour. In group A, the reduction was excellent in 20 cases (83.3%), good in 2 cases (8.3%), and poor in 2 cases (8.3%), and the excellent and good rate was 91.7%. Four posterolateral fracture fragments were not completely fixed (16.7%). The knee Rasmussen score was 22–28 (average 26.8), and knee range motion was 100–120° (average115.5°). In group B, the reduction was excellent in 6 cases (27.3%), good in 12 cases (54.5%), and poor in 4 cases (18.2%), and the excellent and good rate was 81.8%. Eight posterolateral fracture fragments were not completely fixed (36.4%). The Rasmussen score was 20–28 (average 23.5), and the knee range motion was 95–115° (average 106.6°) in group B. The excellent and good rate of reduction had no significant difference (*P* > 0.05) between the two groups, but the excellent rate of reduction had a significant difference (*P* < 0.001). The unfixed rate of the posterolateral fragment showed no significant difference nor did the Rasmussen score or knee range motion (*P* > 0.05) (Table 2).

**Table 2:** Clinical results

	Group A	Group B	Statistics analysis
Cases	24	22	
Operation time (min)	124	175	*P* < 0.05
Blood loss (ml)	118	190	*P* < 0.05
Bone healing time (months)	4.58	5.54	*P* > 0.05
Knee Rasmussen score	26.8	23.5	*P* > 0.05
Knee range motion	115.5°	106.6°	*P* > 0.05
Fracture reduction evaluation			
Excellent	20 (83.3%)	6 (27.3%)	*P* < 0.001
Good	2 (8.3%)	12 (54.5%)	
Poor	2 (8.3%)	4 (18.2%)	
Posterolateral fragments unfixed	4 (16.7%)	8 (36.4%)	*P* > 0.05
Arthroscopic treatment	6	6	

### Complications

One patient in group B suffered postoperative wound infection. The lateral plate was removed, and negative vacuum drainage was performed. After three sessions of negative vacuum drainage, the wound healed, and finally, the fracture healed. No internal fixation failure, no postoperative compartment syndrome, and no other postoperative complications occurred.

## Discussion

Among tibial plateau fractures, posterolateral tibial plateau fracture had received increasing attention. The traditional anterolateral approach could not expose the articular surface directly, which lead to poor reduction [18] and unreliable fixation. The posterior approaches were effective, but they had shortcomings such as the possibility of neurovascular injury, posterior soft tissue injury, and inconvenient operation [19, 20]. Therefore, the extended anterolateral approach had recently been applied in the treatment of the posterolateral fracture. The combination of the anterolateral approach and posterior inverted L-shaped approach in the treatment of Schatzker type II fracture with posterolateral fracture had obtained good clinical results [21, 22], but a single extended anterolateral approach also could get similar results.

In our series, if the posterolateral tibial plateau fracture was relatively simple, we could obtain a satisfactory fracture reduction by reducing the lateral tibial cortex and the articular surface under direct vision. However, if the posterolateral fracture was comminuted, sometimes it was difficult to judge whether the posterolateral fracture was reduced under direct vision. There might be some posterolateral fracture collapse or rotation existed. Neither direct vision nor C-arm X-ray could find the malreduction easily. Arthroscopy could help us to judge the reduction quality. We could see all posterolateral fracture fragments by arthroscopy and reduce the posterolateral fracture under the arthroscopic monitor with little surgical damage to confirm all fracture fragments were reduced. If a patient also had meniscus or cruciate ligament injury, it could be treated at the same time [23]. However, there was a certain learning curve in arthroscopy, which prolonged the operation time and increased the cost of treatment. In addition, arthroscopy was difficult to use in some comminuted tibial plateau fractures because of severe fluid drainage from comminuted metaphyseal fracture site. We thought that the arthroscopy treatment was more helpful in the relatively comminuted/complex posterolateral tibial plateau fracture to make a clear visualization of the articular surface and good reduction.

The traditional anterolateral approach could not place the plate across the fibular head, leading to one-screw or no-screw fixation of the posterolateral fracture. Sassoon et al.’s study [24] showed that the AP distance that was unsupported and located behind the posterior-most rafting screw averaged 16 mm, which represented 42% of the entire AP depth of the lateral plateau, and the mechanical strength of the single screw fixation was relatively limited [25]. Therefore, the traditional anterolateral approach was often unable to achieve a reliable fixation for the posterolateral fracture. In the extended anterolateral approach, at least two screws could fix the posterolateral fracture by placing the locking plate across over the fibular head. Biomechanical tests showed that the mechanical strength obtained by this method greatly increased [26]. The majority of patients obtained reliable internal fixation by the lateral locking plate with rafting screws. However, when the posterolateral fracture was comminuted, some fracture fragment might not be fixed by rafting screws. In our patients, the unfixed rate of the posterolateral fragment was relatively higher in group B. Sun et al. [27] introduced the “magic screw” technique to enhance the lateral rafting plate fixation to posterolateral plateau fracture, and we thought it might be a good choice in extended anterolateral approach which could provide similar biomechanical stability close to a posterior buttress plate.

We divided our patients into two groups, Schatzker type II and type V/VI tibial plateau fracture patients, mainly based on the fracture severity and surgical difficulty, to compare the treatment of the extended anterolateral approach in relatively simple fractures vs. complex fractures. The results showed that in relatively simple type II tibial plateau fractures, the operation time and the amount of bleeding were significantly less and the excellent fracture reduction rate was significantly higher than that of type V/VI tibial plateau fractures. Though the excellent and good rate of reduction and posterolateral fracture fixation rate made no significant difference, they were better in group A. These results confirmed that it was easier to make fracture reduction and fixation in simple tibial plateau fracture by the extended anterolateral approach. The operation was easier in the simple tibial plateau fracture. However, the similar results of the knee functional score and knee range motion in two groups showed that though the operation was easier in simple tibial plateau fracture, the extended anterolateral approach treatment could obtain satisfactory clinical results in either simple or complex tibial plateau fractures.

In our cases, only one patient in group B had a postoperative infection. The causes of postoperative infection were related to severe original injury of soft tissue, double incisions, and long operation time (300 min). Compared with the previous literature, the incidence of complications was less [28]. There is no possibility of posterior soft tissue dissection or neurovascular injury which might occur in the posterior approaches. The extended anterolateral approach could effectively reduce the incidence of intraoperative and postoperative complications.

Therefore, the extended anterolateral approach assisted with arthroscopy treatment could obtain satisfactory clinical results both in simple/complex tibial plateau fracture with posterolateral plateau fracture. In simple tibial plateau fracture, it was easier to finish the operation with the less operative time, less bleeding, and better excellent fracture reduction rate. But these differences did not influence the final clinical results compared to complex tibial plateau fracture.

### Limitations

This was a retrospective study, and all patients were from our hospital, so the collected data had a greater chance of bias. MRI examinations were not done in most patients; thus, we might have failed to accurately describe the ligaments of the knee joint and the soft tissue injury. Finally, we had no control group for comparison with other approaches.

## Conclusion

The extended anterolateral approach could obtain similar satisfactory clinical results in simple/complex tibial plateau fracture with posterolateral tibial plateau fracture. It seemed that easier operation, better posterolateral fracture reduction, and fixation occurred in relative simple fracture from our cases.
